# 20D-Dynamic Representation of Protein Sequences Combined with *K*-means Clustering

**DOI:** 10.2174/0113862073359729250220131623

**Published:** 2025-02-26

**Authors:** Dorota Bielińska-Wąż, Piotr Wąż, Agata Błaczkowska

**Affiliations:** 1 Department of Radiological Informatics and Statistics, Medical University of Gdańsk, 80-210 Gdańsk, Poland;; 2 Department of Nuclear Medicine, Medical University of Gdańsk, 80-210 Gdańsk, Poland

**Keywords:** Alignment-free methods, graphical bioinformatics, 20D-dynamic representation of protein sequences, *K-*means clustering, descriptors, machine learning

## Abstract

**Objective:**

The objective of this research is to demonstrate that alignment-free bioinformatics approaches are effective tools for analyzing the similarity and dissimilarity of protein sequences. All numerical parameters representing sequences are expressed analytically, ensuring precision, clarity, and efficient processing, even for large datasets and long sequences. Additionally, a novel approach for identifying previously unknown virus strains is introduced.

**Methods:**

A novel approach is proposed, integrating the unique features of our newly developed method, the 20D-Dynamic Representation of Protein Sequences, with the *K-*means clustering algorithm. The sequences are represented as clouds of material points in a 20-dimensional space (20D-dynamic graphs), with their spatial distribution being unique to each protein sequence. The numerical parameters, referred to as descriptors in molecular similarity theory, represent quantities characteristic of dynamic systems and serve as input data for the *K-*means clustering algorithm.

**Results:**

Examples of the application of the approach are presented, including projections of the 20D-dynamic graphs onto 3D spaces, which serve as a visual tool for comparing sequences. Additionally, cluster plots for the analyzed sequences are provided using the proposed method.

**Discussion:**

Combining the 20D-Dynamic Representation of Protein Sequences with an unsupervised machine learning algorithm (*K*-means clustering) enhances its scalability. This approach is applicable to large datasets without restrictions on sequence length.

**Conclusion:**

It has been demonstrated that the 20D-Dynamic Representation of Protein Sequences, combined with the *K-*means clustering algorithm, successfully classifies subtypes of influenza A virus strains.

## 1 INTRODUCTION

Recently, the rapid expansion of biomedical experimental data in databases has driven the creation of mathematical techniques for representing these extensive and intricate datasets [1, 2]. Among these approaches are the alignment-free bioinformatics methods (for a review, see [3]). These methods, designed to compare biological (DNA/RNA/protein) sequences integrate concepts from mathematics, computer science, physics, biology, and chemistry, and are known for their computational efficiency [4, 5]. As a result of the interdisciplinary integration of ideas from different scientific fields in the design of new bioinformatics alignment-free approaches, articles on this topic can be found in journals of mathematics [6], computer science [1], physics [7], biology [4, 5], and chemistry [8]. For instance, Gupta *et al.* introduced a method to analyze protein sequence similarity using Chou’s pseudo amino acid composition in its general form [9]. Li *et al.* created an alignment-free method based on pseudo-Markovian transition probabilities among amino acids to compare protein sequences [10]. Saw *et al.* proposed a fuzzy integral-based alignment-free approach for protein sequence similarity analysis [11]. Zhao *et al.* proposed a method for protein sequence comparison that relies on the physicochemical properties of amino acids [12]. Some alignment-free bioinformatics methods are based on chaos game representation [13].

A subset of alignment-free methods, known as Graphical Representations of Biological Sequences, offers both numerical and visual tools for sequence analysis and is used for the similarity analysis of DNA, RNA, and protein sequences. A review covering developments up to 2013 can be found in the article by Randić *et al.* [14]. One of the earliest two-dimensional graphical representations of DNA sequences was created by Nandy [15], with subsequent advancements made by Nandy *et al.* [16]. Randić *et al.* developed a graphical representation of DNA sequences using four horizontal lines [17], and also introduced a protein representation that relies on generalized star graphs, where a central vertex of maximal degree connects to other vertices with degrees one or two [18]. Cao *et al.* transformed DNA sequences into four three-dimensional graphical representations, focusing on the chemical properties of neighboring dual nucleotides [19]. Jafarzadeh and Iranmanesh developed a 3D graphical representation of DNA sequences that relies on codons [20]. Mu *et al.* introduced the 3D-PAF curve for representing protein sequences, which accounts for the cumulative frequencies of adjacent amino acids [21]. We created a spectral graphical representation of DNA sequences [22]. Zhang and Wen developed a two-dimensional graphical representation of protein sequences based on two physicochemical properties of amino acids: isoelectric point and hydrophobicity index [23]. Mahmoodi-Reihani *et al.* considered twelve physicochemical properties of amino acids to create a new two-dimensional graphical representation of protein sequences [24], while Li *et al.* modeled protein sequences as time series in an eleven-dimensional space [25]. Qi *et al.* proposed a new graphical representation of protein sequences that relies on 3-ary Huffman coding [26]. A recent review of graphical representations can be found in the article by Nandy, which describes more approaches aimed at analyzing biological sequence similarity/dissimilarity [27].

The challenge of similarity analysis is inherently interdisciplinary and provides valuable insights across various scientific fields [28]. For example, we have explored the similarity of diverse objects such as molecular spectra [29], groups of individuals [30], and DNA sequences [31], focusing on different aspects of their similarity.

Complex objects may appear very similar based on one feature, yet differ significantly when another feature is considered. Each computational method highlights distinct aspects of object similarity, making the development of computational approaches crucial for advancing this area of study.

The current work is a continuation of our previous studies [32, 33]. In this work, we propose a novel approach that combines the bioinformatics approach we introduced, known as the 20D-Dynamic Representation of Protein Sequences [32], with the *K-*means clustering algorithm [34, 35].

## 2 MATERIALS AND METHODS

In the 20D-Dynamic Representation of Protein Sequences, each sequence is represented as a graph constructed using the concept of a shift ("walk") in a 20-dimensional space, starting from the origin of the coordinate system. Each step in this algorithm is defined by a unit vector representing the relevant amino acid in the sequence, with a "material point" ("point mass") with mass positioned at the end of each vector. The initial shift corresponds to the first amino acid in the sequence, while the next shift represents the second amino acid and begins at the end of the first vector. This process is repeated for each subsequent amino acid until the entire sequence is represented. As a result, the 20D-dynamic graph is created, comprising material points in 20-dimensional space. The distribution of these material points is determined by the sequence represented by the graph. Details on the assignment of the 20-dimensional coordinate system axes to the amino acids (*i.e.*, the representation of amino acids by unit basis vectors in 20D space) are provided in Table **1**. Changing the assignment of the 20-dimensional coordinate system axes to amino acids (*i.e.*, representing amino acids with different vectors in 20D space) does not affect the similarity relations between sequences. However, this change must be applied consistently across all sequences.

As an example, Alanine (A) is depicted by the unit vector (1, 0, 0, 0, 0, 0, 0, 0, 0, 0, 0, 0, 0, 0, 0, 0, 0, 0, 0, 0), whereas Tyrosine (Y) is represented by (0, 0, 0, 0, 0, 0, 0, 0, 0, 0, 0, 0, 0, 0, 0, 0, 0, 0, 0, 1).

To visually represent protein sequences, we introduced projections of 20D-dynamic graphs onto 2D or 3D spaces. The mass distributions in these 2D/3D spaces offer insights into the positions of two or three amino acids within the sequence, facilitating graphical comparisons. Examples of 20D-dynamic graphs and their projections for model sequences were presented in our previous articles [32, 33].

To numerically characterize the 20D-dynamic graphs, we employ values equivalent to those utilized in dynamics. In dynamics, common descriptors for the mass distribution within a rigid body include the center of mass and the moment of inertia. The moment of inertia about a specific axis describes the distribution of mass around that axis. Moments of inertia related to the principal axes are known as the principal moments of inertia. This concept can be extended to describe distributions of other quantities. Specifically, by assigning a "mass" to each point in the 20D-dynamic graph, we can define a moment of inertia for the graph, serving as an analogy to the traditional moment of inertia.

The coordinates of the center of mass for a 20D-dynamic graph are determined as follows:


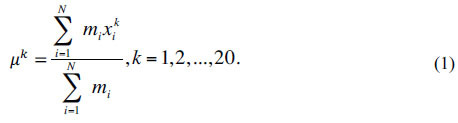



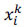
 are the coordinates of the material points with masses *m_𝑖_*. The number of the material points in the 20D-dynamic graph (*N*) is the length of the sequence. Since each material point has a mass of one, the total mass of the sequence is equivalent to its length, *i.e.*, 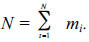


Finally,


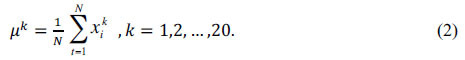


The moment of inertia tensor in 20D space is represented by the matrix


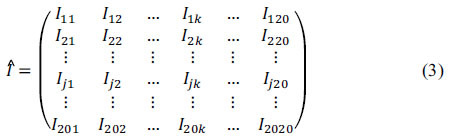


with the matrix elements


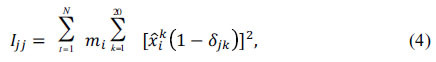


and


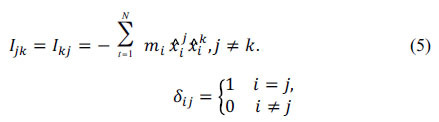


is the Kronecker-Delta. 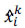 denote the coordinates of *m_𝑖_* in the Cartesian coordinate system, where the origin is placed at the center of mass


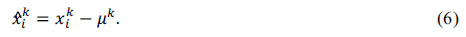


The eigenvalue problem for the inertia tensor with its corresponding eigenvalues *I_𝑖_* and the eigenvectors 𝜔*_k_* is formulated as follows


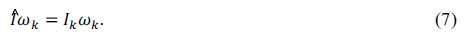


The eigenvalues are obtained by solving the twentieth-order secular equation


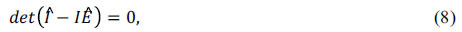


where 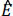 is 20x20 unit matrix. The eigenvalues *I_k_,k* = 1,2...,20 are referred to as the *principal moments of inertia*.

In this study, we introduce descriptors for characterizing protein sequences, analogous to those previously used to describe DNA/RNA sequences [36, 37], defined as:


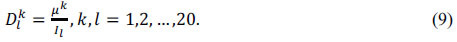


In the present work, the descriptors of the 20D-Dynamic Representation of Protein Sequences are employed as input data for the *K-*means clustering algorithm.

Alignment-free bioinformatics approaches are designed to characterize 20 amino acids. The proposed method can be easily adapted to higher dimensions if a sequence contains more than 20 amino acids. The core structure of the algorithm remains unchanged, with new basis vectors introduced using the same process as for the original 20 dimensions. By incorporating selenocysteine and pyrrolysine, the representation expands to a 22D-dynamic graph. For numerical characterization, the descriptors in 22D space should be applied. As a result, summation limits throughout the method must be updated to account for the expanded dimensionality.

The proposed approach is applied to analyze the similarity of sequences studied by Hou *et al.* (subtypes of the influenza A virus sequences), who introduced Dynamic Time Warping, another alignment-free bioinformatics method [38]. The sequence data are presented in Table **2**. All sequences were obtained from the NCBI database. One sequence, A/goose/Czech_Republic/1848-K9/2009(H7N9), which contains an unidentified amino acid and was considered by Hou *et al.,* has been excluded from our analysis.

## 3 RESULTS AND DISCUSSION

The influenza A virus poses a significant threat to both humans and animals. These viruses are classified into various subtypes based on their surface proteins, hemagglutinin, and neuraminidase. To date, 18 hemagglutinin serotypes (H1 to H18) and 11 neuraminidase serotypes (N1 to N11) have been identified. Influenza A viruses have caused widespread epidemics in both humans and animals, with some of the most lethal strains being H1N1, H3N2, H5N1, and H7N9 [39-42].

The calculations were performed using functions and procedures from the R environment [35, 43, 44].

Examples of 3D graphs for several analyzed sequences are shown in Fig. (**1**): GLV-graphs (top left), ALM-graphs (top right), AEF-graphs (middle left), AFG-graphs (middle right), AST-graphs (bottom left), and AWY-graphs (bottom right). For comparison, the corresponding 3D graphs for NADH dehydrogenase subunit 6 (ND6) protein sequences, studied in our previous work [32], are displayed in Fig. (**2**). The ranges and shapes of the graphs differ between the two figures, with smaller yet noticeable differences even within the same type of sequences. These subtle differences can be effectively represented numerically.

Let us note that the projection of the 20D-dynamic graph into a 3D space is achieved by selecting three specific coordinates of the material points. For example, as shown in Table **1**, the axes numbered 6, 10, and 18 correspond to the amino acids G, L, and V. Consequently, in GLV-graphs, the coordinates of the material points are taken from axes 6, 10, and 18.

Tables **3** through 6 present the descriptors of the 20D-Dynamic Representation of Protein Sequences for the analyzed sequences. The coordinates of the centers of mass were calculated using equation (2), while the principal moments of inertia were determined using equation (8). As mentioned earlier, these parameters describe the mass distribution within the rigid body, and in our case, they characterize the shapes of the graphs corresponding to the sequences. The coordinates of the centers of mass are provided in Tables **3** and **4**, while the principal moments of inertia are detailed in Tables **5** and **6**. The descriptor values vary between sequences.

A clear visualization of the similarity relations is provided by cluster plots obtained using a combination of the 20D-Dynamic Representation of Protein Sequences and the *K-*means clustering algorithm.

**Fig. (3):**
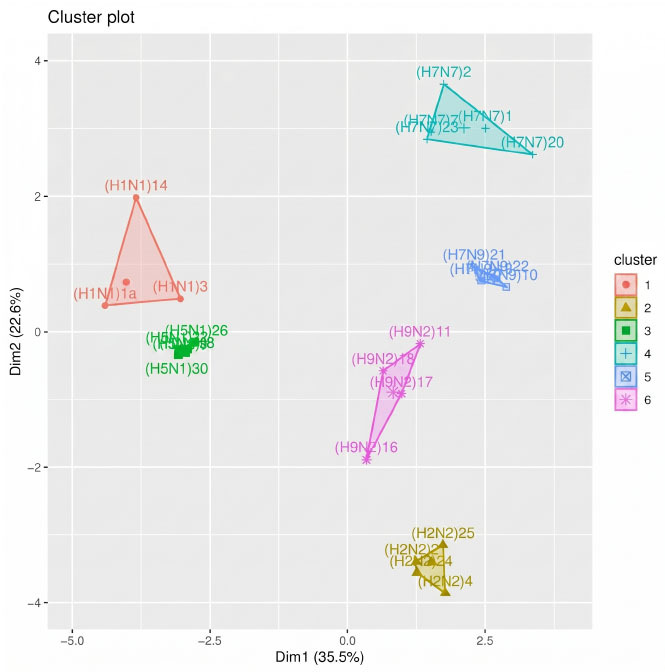
Cluster plot obtained using *K-*means clustering with 𝜇^𝑘^ (Eq. 2) as input data.

**Fig. (4):**
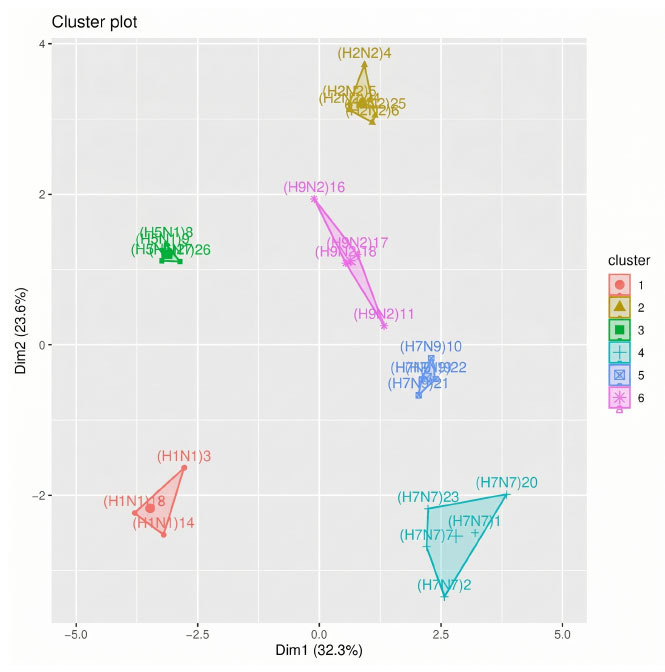
Cluster plot obtained using *K-*means clustering with 𝐷^𝑘^_𝑙_ (Eq. 9) as input data.

Figs. (**3** and **4**) display cluster plots generated with *K-*means clustering with the descriptors of the 20D-dynamic graphs as input data: (Fig. **3**) and (Fig. **4**).

The number of clusters was set to 6 based on the information about the change in the total sum of squares of pairwise distances and the change in the growth of the sum of squares relative to the total sum of squares. To make the result more stable, 25 different initial assignments (for the cluster center coordinates) were tested each time, and the one with the smallest internal variability within the cluster was selected. The maximum number of iterations was set to 100.

As a result of our analysis, we determined the distances between points in the 20-dimensional space. To visualize the obtained result in a 2-dimensional space, we used Principal Component Analysis (PCA), which involves finding new variables (combinations of the old variables) that maximize the variance. Dim1 and Dim2 are the new variables (combinations of the used variables) with the highest variance.

The calculation of the Silhouette score (Fig. **3**) shows that the observations from the H7N9 cluster are most similar to the assigned cluster (average silhouette width: 0.72) compared to the other clusters, while the lowest score was obtained for the H9N2 cluster (average silhouette width: 0.22). For the H5N1, H2N2, H1N1, and H7N7 clusters, the average values of the score were as follows: 0.7, 0.63, 0.46, and 0.31.

The calculation of the Silhouette score (Fig. **4**) indicates that the observations from the H5N1 and H7N9 clusters are most similar to the assigned cluster (average silhouette width: 0.72) compared to the other clusters, while the lowest score was obtained for the H9N2 cluster (average silhouette width: 0.2). For the H2N2, H1N1, and H7N7 clusters, the average values of the score were as follows: 0.64, 0.58, and 0.34.

Both cluster plots correctly classify the sequences: points corresponding to different subtypes H1N1, H5N1, H9N2, H2N2, H7N9, and H7N7 are positioned in distinct regions of the plots. The structures of the two cluster plots differ, suggesting that each set of descriptors reveals unique features of the similarity between sequences.

Another common way to visualize results is through a dendrogram, a diagram representing a tree structure. Visualizations of the data considered can be found in the study by Hou *et al.* [38], where both traditional methods and Dynamic Time Warping (an alignment-free bioinformatics approach introduced by Hou *et al*.) are employed. Figs. (**5** and **6**) present dendrograms generated using our method: Fig. (**5**) illustrates the results based on the coordinates of the centers of mass (Eq. 2) and the descriptors D (Eq. 9). Notably, Hou *et al.* observed low similarity values between H7N7 and H7N9 using their method, positioning these elements far apart in their tree. In contrast, our dendrograms position H7N7 and H7N9 close to each other, aligning more closely with the traditional method.

## CONCLUSION

In summary, the 20D-Dynamic Representation of Protein Sequences proves to be a powerful tool for both visual and numerical analysis of sequence similarities. It is essential to develop a diverse array of bioinformatics methods, as each can uncover different facets of similarity. It has been shown that the 20D-Dynamic Representation combined with the *K-*means clustering algorithm effectively classifies influenza A virus subtypes. Developing tools to characterize unidentified viruses is especially crucial.

New, accurate mathematical approaches for studying the similarity of primary protein structures are also essential for understanding more complex structural phenomena, such as those explored by Beaudoin *et al.* [45].

The urgency of advancing bioinformatics tools has grown significantly, particularly in light of recent global health challenges.

## STUDY LIMITATIONS

The proposed method offers analytical descriptors and computational efficiency for large-scale datasets, including long sequences. While the 20D-Dynamic Representation of Protein Sequences combined with an unsupervised machine learning algorithm (*K*-means clustering) demonstrates robust classification performance, further validation across diverse biological contexts may strengthen its generalizability. Theoretical scalability is unrestricted, but empirical testing under varied conditions remains an area for future exploration.

## Figures and Tables

**Fig. (1):**
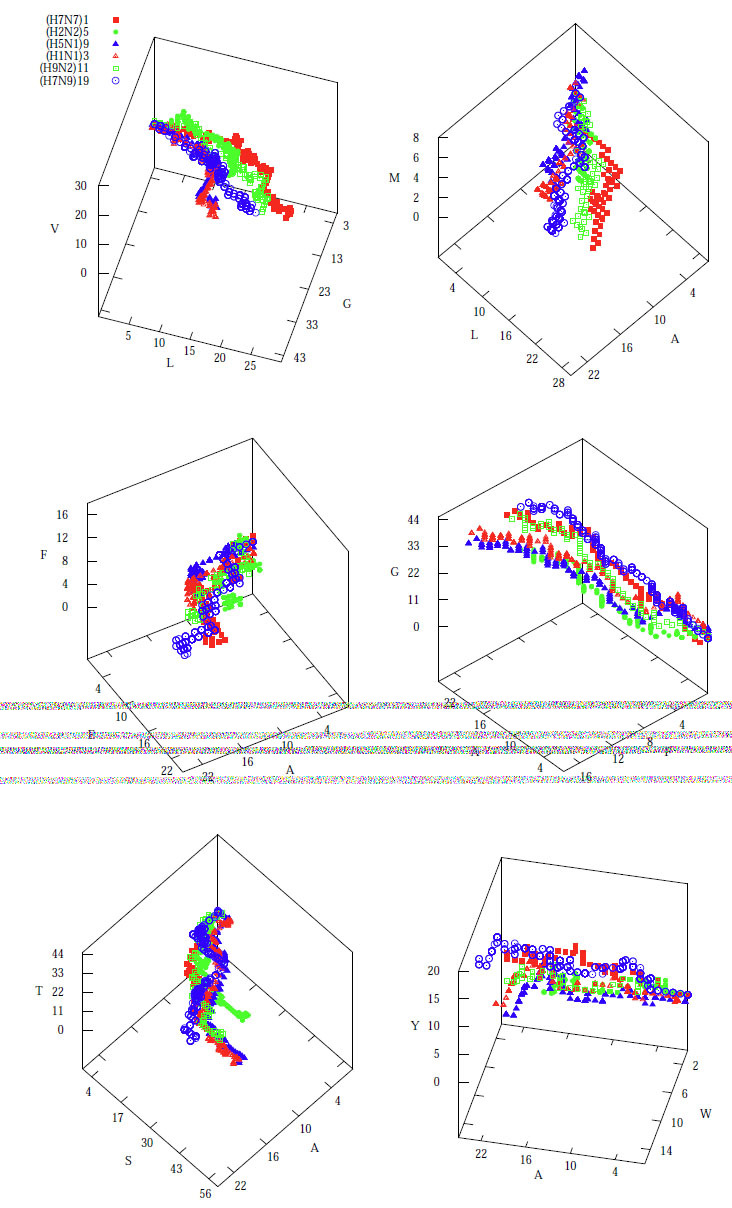
3D graphs representing influenza A virus sequences.

**Fig. (2):**
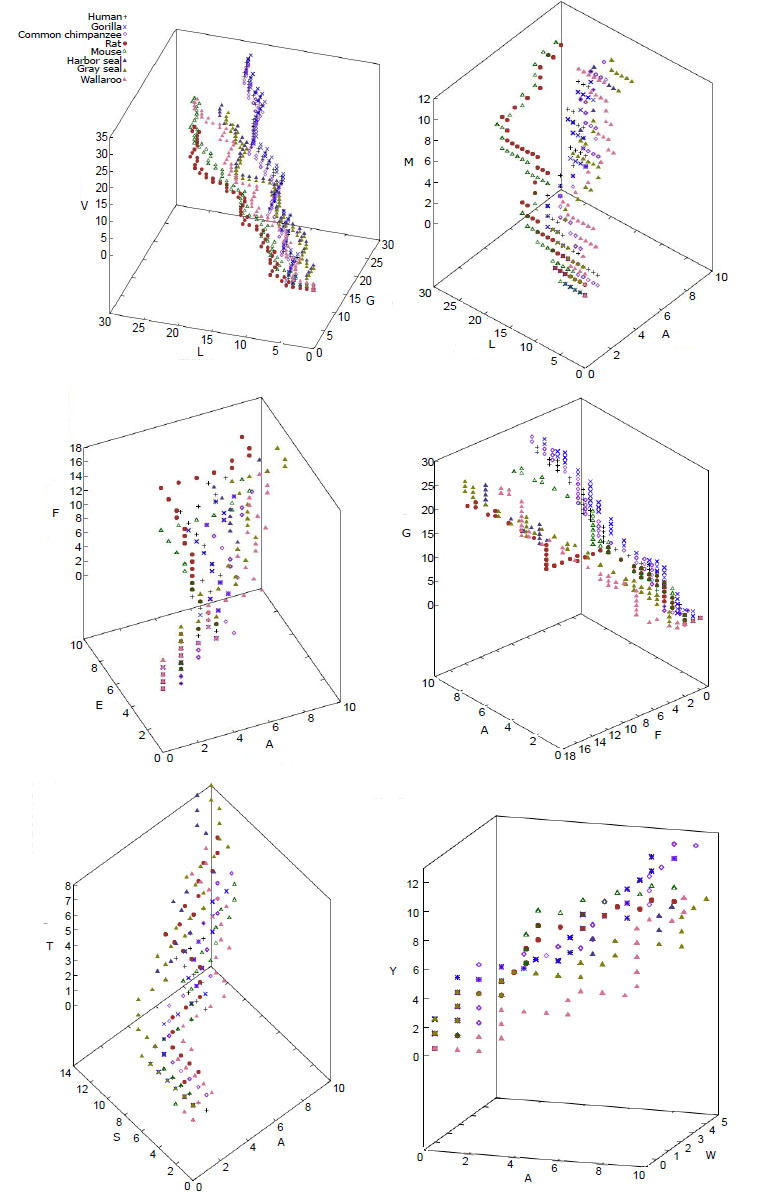
3D graphs representing ND6 protein sequences. The panels match those in Figure **1**.

**Fig. (5):**
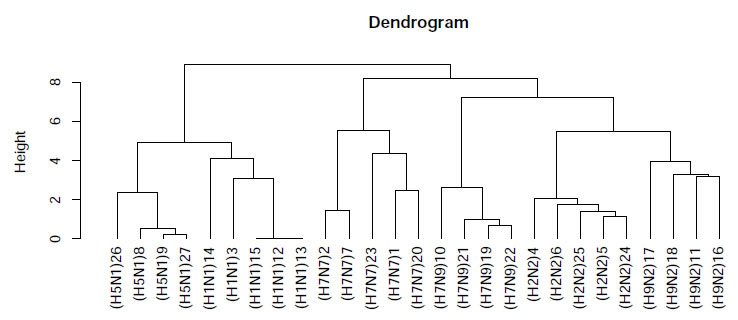
Dendrogram using 𝜇^𝑘^ (Eq. 2) as input data.

**Fig. (6):**
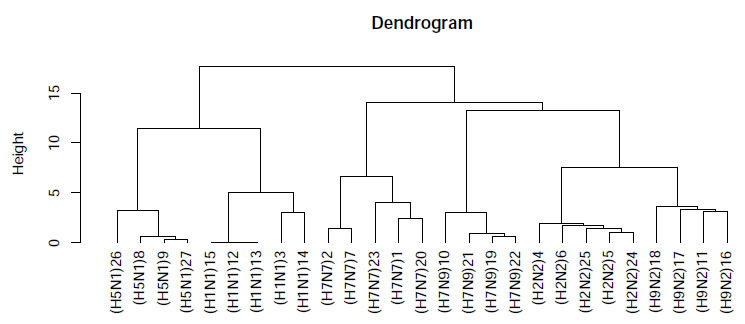
Dendrogram using 𝐷^𝑘^_𝑙_ (Eq. 9) as input data.

**Table 1:** Representation of the amino acids in the 20-dimensional space.

**Axis No.**	**Amino Acid**	**One-letter Symbol**
1.	Alanine	A
2.	Cysteine	C
3.	Aspartic acid	D
4.	Glutamic acid	E
5.	Phenylalanine	F
6.	Glycine	G
7.	Histidine	H
8.	Isoleucine	I
9.	Lysine	K
10.	Leucine	L
11.	Methionine	M
12.	Asparagine	N
13.	Proline	P
14.	Glutamine	Q
15.	Arginine	R
16.	Serine	S
17.	Threonine	T
18.	Valine	V
19.	Tryptophan	W
20.	Tyrosine	Y

**Table 2:** Sequence data.

**Notation**	**Sequence Name**	**Accession No.**	**N**
(H7N7)1	A/fowl/Weybridge(H7N7)	AAA43425.1	471
(H7N7)2	A/equine/Prague/1/1956(H7N7)	AAC57418.1	469
(H1N1)3	A/Duck/Ohio/118C/93(H1N1)	AAF77041.1	469
(H2N2)4	A/England/1/1961(H2N2)	AAO46220.1	469
(H2N2)5	A/California/1/1966(H2N2)	AAO46235.1	469
(H2N2)6	A/Georgia/1/1967(H2N2)	AAO46244.1	469
(H7N7)7	A/equine/Santiago/77(H7N7)	AAQ90293.1	469
(H5N1)8	A/Cygnus_olor/Italy/742/2006(H5N1)	ABF50822.1	449
(H5N1)9	A/cat/Germany/R606/06(H5N1)	ABF61763.1	449
(H7N9)10	A/blue-winged_teal/Ohio/566/2006(H7N9)	ABS89412.1	470
(H9N2)11	A/chicken/Iran/B263/2004(H9N2)	ACD47112.1	469
(H1N1)12	A/GuangzhouSB/01/2009(H1N1)	ACR49238.1	469
(H1N1)13	A/Beijing/4/2009(H1N1)	ACR67256.1	469
(H1N1)14	A/Nagasaki/07N020/2008(H1N1)	ADC45738.1	470
(H1N1)15	A/California/04/2009(H1N1)	AEE69012.1	469
(H9N2)16	A/chicken/China/AH-10-01/2010(H9N2)	AEE73586.1	466
(H9N2)17	A/canine/Guangxi/1/2011(H9N2)	AEK07935.1	469
(H9N2)18	A/chicken/Hubei/01-MA01/1999(H9N2)	AEO92432.1	466
(H7N9)19	A/tree_sparrow/Shanghai/01/2013(H7N9)	AGW82590.1	465
(H7N7)20	A/lesser_white-fronted_goose/HuNan/412-3Y/2010(H7N7)	AIW60686.1	471
(H7N9)21	A/chicken/Dongguan/1096/2014(H7N9)	AJJ96855.1	465
(H7N9)22	A/chicken/Quzhou/2/2015(H7N9)	AKI82227.1	465
(H7N7)23	A/blue_winged_teal/Louisiana/A00557206/2009(H7N7)	ALT67567.1	470
(H2N2)24	A/Adachi/2/1957(H2N2)	BAD16637.1	469
(H2N2)25	A/Berkeley/1/1968(H2N2)	BAD16641.1	469
(H5N1)26	A/muscovy_duck/Vietnam/LBM66/2011(H5N1)	BAM36161.1	449
(H5N1)27	A/bar-headed_goose/Qinghai/1/2005(H5N1)	BAM85828.1	449

**Table 3:** Coordinates of the centers of mass for the sequences listed in Table **2**.

**Notation**	**𝜇^1^**	**𝜇^2^**	**𝜇^3^**	**𝜇^4^**	**𝜇^5^**	**𝜇^6^**	**𝜇^7^**	**𝜇^8^**	**𝜇^9^**	**𝜇^10^**
(H7N7)1	10.69	9.465	7.414	10.45	4.503	19.06	5.255	17.85	11.20	19.16
(H7N7)2	10.25	9.043	7.220	11.06	5.277	16.16	5.770	21.71	13.14	16.00
(H1N1)3	11.67	9.373	7.742	9.211	6.388	19.38	3.876	26.54	9.094	10.70
(H2N2)4	10.86	11.67	13.19	12.16	6.586	14.86	6.597	22.40	8.156	12.65
(H2N2)5	9.096	11.67	12.66	11.33	6.586	15.25	6.597	22.93	9.648	12.65
(H2N2)6	10.06	11.67	12.66	11.33	6.881	15.25	6.597	21.96	9.648	12.65
(H7N7)7	9.844	9.043	7.844	11.24	5.277	15.31	5.770	22.91	12.48	16.00
(H5N1)8	11.20	8.808	8.178	8.815	7.555	19.82	4.706	20.71	9.539	11.09
(H5N1)9	11.20	8.808	8.477	8.815	7.555	19.52	4.706	20.71	9.539	11.09
(H7N9)10	13.92	10.84	7.340	11.50	3.000	16.18	5.855	21.12	7.849	12.91
(H9N2)11	11.18	11.67	9.503	10.09	5.066	17.19	5.680	21.50	11.45	15.99
(H1N1)12	9.640	9.373	7.249	8.678	7.232	19.49	3.143	25.69	8.923	10.35
(H1N1)13	9.640	9.373	7.249	8.678	7.232	19.49	3.143	25.69	8.923	10.35
(H1N1)14	11.22	8.419	8.757	8.345	6.570	20.88	4.332	27.73	11.42	10.84
(H1N1)15	9.640	9.373	7.249	8.678	7.232	19.49	3.143	25.69	8.923	10.35
(H9N2)16	12.28	11.73	9.586	10.44	6.047	15.45	5.268	23.24	10.97	13.59
(H9N2)17	13.64	11.67	9.495	9.881	5.424	18.20	7.458	21.85	9.279	15.33
(H9N2)18	11.78	11.73	8.650	9.966	6.047	16.38	6.575	22.10	14.60	13.59
(H7N9)19	11.99	10.92	8.002	10.59	3.032	16.98	5.897	22.25	7.677	12.99
(H7N7)20	11.42	9.006	7.011	12.72	4.541	17.48	5.762	17.79	12.01	18.26
(H7N9)21	11.99	10.92	8.002	10.59	3.032	16.98	5.897	22.51	8.512	12.99
(H7N9)22	11.99	10.92	8.002	10.59	3.032	16.98	5.897	21.85	7.677	12.99
(H7N7)23	11.56	9.026	9.417	9.296	4.511	18.34	5.772	15.75	11.91	19.31
(H2N2)24	10.03	11.67	12.35	11.71	6.586	15.70	6.597	22.32	9.243	12.44
(H2N2)25	10.07	11.67	13.06	11.33	6.578	14.86	6.597	21.47	10.72	12.66
(H5N1)26	11.27	8.808	7.733	9.494	7.846	19.33	5.842	23.34	8.581	11.09
(H5N1)27	11.20	8.808	8.178	8.815	7.555	19.82	4.706	20.71	9.539	11.09

**Table 4:** Coordinates of the centers of mass for the sequences listed in Table **2**.

**Notation**	**𝜇^11^**	**𝜇^12^**	**𝜇^13^**	**𝜇^14^**	**𝜇^15^**	**𝜇^16^**	**𝜇^17^**	**𝜇^18^**	**𝜇^19^**	**𝜇^20^**
(H7N7)1	3.830	23.25	8.826	6.807	9.255	18.10	24.09	17.26	2.981	6.561
(H7N7)2	6.036	27.68	8.597	5.908	7.876	18.97	22.25	12.14	2.925	6.994
(H1N1)3	3.823	20.61	10.58	8.209	7.968	27.03	15.49	13.81	6.094	7.426
(H2N2)4	4.578	14.73	11.56	9.403	11.43	20.40	17.54	15.89	4.682	5.659
(H2N2)5	4.578	14.71	11.29	9.582	10.99	20.55	18.03	16.64	4.539	5.659
(H2N2)6	3.627	14.71	10.56	9.582	10.99	20.99	19.95	15.68	4.539	5.659
(H7N7)7	5.646	26.47	8.124	6.699	8.096	20.35	21.23	12.74	2.925	6.994
(H5N1)8	5.746	15.49	10.38	7.461	9.706	26.47	13.45	15.23	5.410	5.234
(H5N1)9	5.746	15.41	10.38	7.461	9.080	27.17	13.45	15.23	5.410	5.234
(H7N9)10	3.489	25.08	11.69	8.172	11.49	19.22	20.03	12.49	4.651	8.691
(H9N2)11	3.872	17.86	9.727	8.787	9.243	21.04	20.42	14.17	4.539	6.013
(H1N1)12	4.716	23.90	9.582	9.951	7.516	27.00	13.89	15.44	6.094	7.158
(H1N1)13	4.716	23.90	9.582	9.951	7.516	27.00	13.89	15.44	6.094	7.158
(H1N1)14	4.806	20.70	8.066	7.413	7.023	26.59	16.30	12.55	6.109	7.426
(H1N1)15	4.716	23.90	9.582	9.951	7.516	27.00	13.89	15.44	6.094	7.158
(H9N2)16	5.185	16.11	10.77	9.682	10.40	22.90	16.54	13.42	4.712	5.187
(H9N2)17	5.256	15.77	9.821	8.186	9.810	22.12	17.54	14.57	4.539	5.154
(H9N2)18	5.185	17.26	10.77	8.004	8.427	20.94	17.13	14.46	4.712	5.187
(H7N9)19	3.170	22.97	11.34	6.966	12.24	21.17	20.49	10.85	4.701	8.763
(H7N7)20	3.830	21.86	9.393	6.807	9.359	19.18	24.27	15.81	2.981	6.512
(H7N9)21	3.170	22.97	11.34	6.966	11.41	21.17	20.49	10.58	4.701	8.763
(H7N9)22	3.170	23.50	11.83	6.966	12.24	20.16	21.46	10.28	4.701	8.763
(H7N7)23	4.838	21.51	6.794	7.587	8.509	20.99	22.32	17.64	3.889	6.526
(H2N2)24	4.663	14.73	11.64	9.320	10.41	20.91	18.03	16.31	4.682	5.659
(H2N2)25	4.578	14.71	11.29	8.778	10.73	20.55	18.90	16.25	4.539	5.659
(H5N1)26	5.200	18.21	9.254	7.461	9.102	26.10	13.10	13.43	5.410	4.410
(H5N1)27	5.746	15.41	10.38	7.461	9.080	27.17	13.45	15.23	5.410	5.234

**Table 5:** Principal moments of inertia for the sequences listed in Table **2**.

**Notation**	***I*_1_/10^4^**	***I*_2_/10^4^**	***I*_3_/10^4^**	***I*_4_/10^4^**	***I*_5_/10^4^**	***I*_6_/10^4^**	***I*_7_/10^4^**	***I*_8_/10^4^**	***I*_9_/10^4^**	***I*_10_/10^4^**
(H7N7)1	54.81	54.81	54.81	54.81	54.81	54.81	54.80	54.80	54.80	54.80
(H7N7)2	53.78	53.78	53.78	53.78	53.77	53.77	53.77	53.77	53.77	53.76
(H1N1)3	55.07	55.07	55.07	55.07	55.07	55.06	55.06	55.06	55.06	55.06
(H2N2)4	53.98	53.98	53.98	53.98	53.98	53.98	53.98	53.97	53.97	53.97
(H2N2)5	54.05	54.05	54.04	54.04	54.04	54.04	54.04	54.04	54.04	54.03
(H2N2)6	53.93	53.93	53.93	53.93	53.93	53.93	53.93	53.93	53.92	53.92
(H7N7)7	53.30	53.30	53.30	53.30	53.30	53.29	53.29	53.29	53.29	53.29
(H5N1)8	47.48	47.48	47.48	47.48	47.48	47.48	47.48	47.48	47.47	47.47
(H5N1)9	47.65	47.65	47.65	47.65	47.65	47.65	47.65	47.65	47.64	47.64
(H7N9)10	52.85	52.85	52.85	52.85	52.85	52.84	52.84	52.84	52.84	52.83
(H9N2)11	54.44	54.44	54.44	54.44	54.44	54.44	54.43	54.43	54.43	54.42
(H1N1)12	56.36	56.36	56.36	56.36	56.36	56.36	56.36	56.35	56.35	56.35
(H1N1)13	56.36	56.36	56.36	56.36	56.36	56.36	56.36	56.35	56.35	56.35
(H1N1)14	53.88	53.88	53.88	53.88	53.88	53.87	53.87	53.87	53.87	53.87
(H1N1)15	56.36	56.36	56.36	56.36	56.36	56.36	56.36	56.35	56.35	56.35
(H9N2)16	53.25	53.25	53.25	53.24	53.24	53.24	53.24	53.24	53.24	53.24
(H9N2)17	54.56	54.56	54.56	54.56	54.55	54.55	54.55	54.55	54.55	54.55
(H9N2)18	52.66	52.66	52.66	52.66	52.66	52.65	52.65	52.65	52.65	52.65
(H7N9)19	51.90	51.90	51.90	51.90	51.90	51.90	51.89	51.89	51.89	51.89
(H7N7)20	54.93	54.93	54.93	54.93	54.93	54.93	54.93	54.93	54.92	54.92
(H7N9)21	51.89	51.89	51.89	51.89	51.89	51.89	51.88	51.88	51.88	51.88
(H7N9)22	51.84	51.84	51.84	51.84	51.83	51.83	51.83	51.83	51.83	51.82
(H7N7)23	53.64	53.64	53.64	53.63	53.63	53.63	53.63	53.63	53.63	53.62
(H2N2)24	54.35	54.35	54.35	54.34	54.34	54.34	54.34	54.34	54.34	54.34
(H2N2)25	53.82	53.82	53.82	53.82	53.82	53.82	53.82	53.82	53.81	53.81
(H5N1)26	47.52	47.52	47.52	47.52	47.52	47.52	47.52	47.52	47.52	47.51
(H5N1)27	47.90	47.90	47.90	47.90	47.90	47.90	47.89	47.89	47.89	47.89

**Table 6:** Principal moments of inertia for the sequences listed in Table **2**.

**Notation**	***I*_11_/10^4^**	***I*_12_/10^4^**	***I*_13_/10^4^**	***I*_14_/10^4^**	***I*_15_/10^4^**	***I*_16_/10^4^**	***I*_17_/10^4^**	***I*_18_/10^4^**	***I*_19_/10^4^**	***I*_20_/10^4^**
(H7N7)1	54.79	54.79	54.78	54.77	54.76	54.73	54.65	54.50	53.97	1.630
(H7N7)2	53.76	53.76	53.75	53.75	53.73	53.69	53.66	53.44	53.15	1.408
(H1N1)3	55.05	55.05	55.03	55.03	55.01	55.00	54.91	54.90	54.43	1.302
(H2N2)4	53.97	53.97	53.96	53.95	53.95	53.87	53.83	53.74	53.14	1.575
(H2N2)5	54.03	54.03	54.02	54.02	54.01	53.94	53.93	53.83	53.20	1.512
(H2N2)6	53.92	53.91	53.91	53.90	53.90	53.83	53.82	53.71	53.11	1.476
(H7N7)7	53.28	53.27	53.27	53.27	53.24	53.18	53.15	52.95	52.69	1.468
(H5N1)8	47.47	47.47	47.45	47.45	47.44	47.43	47.38	47.27	46.95	1.149
(H5N1)9	47.64	47.64	47.62	47.62	47.61	47.60	47.55	47.43	47.15	1.131
(H7N9)10	52.83	52.82	52.82	52.81	52.79	52.74	52.68	52.56	52.31	1.379
(H9N2)11	54.42	54.42	54.41	54.40	54.39	54.35	54.28	54.16	53.62	1.612
(H1N1)12	56.35	56.33	56.33	56.33	56.31	56.27	56.22	56.14	55.86	1.213
(H1N1)13	56.35	56.33	56.33	56.33	56.31	56.27	56.22	56.14	55.86	1.213
(H1N1)14	53.86	53.85	53.85	53.82	53.82	53.80	53.69	53.60	53.20	1.504
(H1N1)15	56.35	56.33	56.33	56.33	56.31	56.27	56.22	56.14	55.86	1.213
(H9N2)16	53.23	53.23	53.22	53.21	53.20	53.16	53.11	53.03	52.41	1.532
(H9N2)17	54.54	54.54	54.53	54.52	54.51	54.46	54.41	54.40	53.76	1.458
(H9N2)18	52.64	52.64	52.64	52.63	52.60	52.57	52.51	52.39	51.85	1.580
(H7N9)19	51.88	51.88	51.87	51.85	51.84	51.79	51.76	51.61	51.54	1.186
(H7N7)20	54.92	54.91	54.91	54.90	54.90	54.84	54.77	54.55	54.22	1.592
(H7N9)21	51.87	51.87	51.86	51.84	51.83	51.79	51.75	51.59	51.55	1.177
(H7N9)22	51.82	51.82	51.81	51.78	51.78	51.74	51.68	51.59	51.43	1.198
(H7N7)23	53.62	53.62	53.61	53.59	53.59	53.55	53.49	53.42	52.98	1.347
(H2N2)24	54.33	54.33	54.32	54.32	54.31	54.26	54.17	54.09	53.47	1.630
(H2N2)25	53.81	53.81	53.80	53.80	53.78	53.71	53.70	53.60	53.01	1.492
(H5N1)26	47.51	47.50	47.50	47.49	47.47	47.46	47.41	47.27	46.95	1.269
(H5N1)27	47.89	47.88	47.87	47.87	47.85	47.85	47.80	47.67	47.37	1.162

## Data Availability

All the data and supporting information is provided within the article.

## ABBREVIATION

PCA: Principal Component Analysis

## References

[1] Du X., Zhu R., Li Y., Anjum A. (2019). Language model-based automatic prefix abbreviation expansion method for biomedical big data analysis.. Future Gener. Comput. Syst..

[2] Lötsch J., Malkusch S., Ultsch A. (2021). Optimal distribution-preserving downsampling of large biomedical data sets (opdisDownsampling).. PLoS One.

[3] Ramanathan N., Ramamurthy J., Natarajan G. (2022). Numerical characterization of DNA sequences for alignment-free sequence comparison – A review.. Comb. Chem. High Throughput Screen..

[4] Anjum N., Nabil R.L., Rafi R.I., Bayzid M.S., Rahman M.S. (2023). CD-MAWS: An alignment-free phylogeny estimation method using cosine distance on minimal absent word sets. IEEE/ACM Trans.. Comput. Biol. Bioinformat..

[5] Wang T., Yu Z.G., Li J. (2024). CGRWDL: Alignment-free phylogeny reconstruction method for viruses based on chaos game representation weighted by dynamical language model.. Front. Microbiol..

[6] Shanan N.A., Lafta H., Alrashid S. (2021). Using alignment-free methods as preprocessing stage to classification whole genomes. Int. J. Nonlin. Analy.. Applica..

[7] Liao B., Xiang Q., Cai L., Cao Z. (2013). A new graphical coding of DNA sequence and its similarity calculation.. Physica A.

[8] Kha Q.H., Ho Q.T., Le N.Q.K. (2022). Identifying snare proteins using an alignment-free method based on multiscan convolutional neural network and pssm profiles.. J. Chem. Inf. Model..

[9] Gupta M.K., Niyogi R., Misra M. (2013). An alignment-free method to find similarity among protein sequences via the general form of Chou’s pseudo amino acid composition.. SAR QSAR Environ. Res..

[10] Li Y., Song T., Yang J., Zhang Y., Yang J. (2016). An alignment-free algorithm in comparing the similarity of protein sequences based on pseudo-markov transition probabilities among amino acids.. PLoS One.

[11] Saw A.K., Tripathy B.C., Nandi S. (2019). Alignment-free similarity analysis for protein sequences based on fuzzy integral.. Sci. Rep..

[12] Zhao Y., Xue X., Xie X. (2019). An alignment-free measure based on physicochemical properties of amino acids for protein sequence comparison.. Comput. Biol. Chem..

[13] Löchel H.F., Heider D. (2021). Chaos game representation and its applications in bioinformatics.. Comput. Struct. Biotechnol. J..

[14] Randić M., Novič M., Plavšić D. (2013). Milestones in graphical bioinformatics.. Int. J. Quantum Chem..

[15] Nandy A. (1994). A new graphical representation and analysis of DNA sequence structure. I: Methodology and application to globin genes.. Curr. Sci..

[16] Nandy A., Dey S., Basak S., Bielińska-Wąż D., Wąż P. (2016). Characterizing the Zika virus genome – A bioinformatics study.. Curr. Computeraided Drug Des..

[17] Randić M., Vračko M., Lerš N., Plavšić D. (2003). Analysis of similarity/dissimilarity of DNA sequences based on novel 2-D graphical representation.. Chem. Phys. Lett..

[18] Randić M., Zupan J., Vikić-Topić D. (2007). On representation of proteins by star-like graphs.. J. Mol. Graph. Model..

[19] Cao Z., Liao B., Li R. (2008). A group of 3D graphical representation of DNA sequences based on dual nucleotides.. Int. J. Quantum Chem..

[20] Jafarzadeh N., Iranmanesh A. (2013). C-curve: A novel 3D graphical representation of DNA sequence based on codons.. Math. Biosci..

[21] Mu Z.C., Li G.J., Wu H.Y., Qi X.Q. (2016). 3D-PAF curve. A novel graphical representation of protein sequences for similarity analysis.. MATCH Commun. Math. Comput. Chem..

[22] Bielińska-Wąż D., Wąż P. (2017). Spectral-dynamic representation of DNA sequences.. J. Biomed. Inform..

[23] Zhang Y., Wen J. (2018). Similarity analysis of protein sequences based on a new graphical representation method.. Commun. Inf. Syst..

[24] Mahmoodi-Reihani M., Abbasitabar F., Zare-Shahabadi V. (2018). A novel graphical representation and similarity analysis of protein sequences based on physicochemical properties.. Physica A.

[25] Li C., Dai Q., He P. (2022). A time series representation of protein sequences for similarity comparison.. J. Theor. Biol..

[26] Qi Z., Ning Y., Huang Y. (2023). Protein sequence comparison method based on 3-ary huffman coding.. Match (Mulh.).

[27] Nandy A. (2022). Mapping biomolecular sequences: Graphical representations - Their origins, applications and future prospects.. Comb. Chem. High Throughput Screen..

[28] Bielińska A., Majkowicz M., Wąż P., Bielińska-Wąż D. (2019). Mathematical modeling: Interdisciplinary similarity studies. In: Numerical Methods and Applications; Nikolov, G.; Kolkovska, N.; Georgiev, K., Eds.; Springer: Cham.

[29] Wąż P., Bielińska-Wąż D. (2013). Moments of inertia of spectra and distribution moments as molecular descriptors.. MATCH Commun. Math. Comput. Chem..

[30] Bielińska A., Wa̧ż P., Bielińska-Wa̧ż D. (2022). A computational model of similarity analysis in quality of life research: An example of studies in poland.. Life (Basel).

[31] Bielińska-Wąż D., Wąż P. (2021). Non-standard bioinformatics characterization of SARS-CoV-2.. Comput. Biol. Med..

[32] Czerniecka A., Bielińska-Wąż D., Wąż P., Clark T. (2016). 20D-dynamic representation of protein sequences.. Genomics.

[33] Bielińska-Wąż D., Wąż P., Błaczkowska A., Mandrysz J., Lass A., Gładysz P., Karamon J. (2024). Mathematical modeling in bioinformatics: Application of an alignment-free method combined with principal component analysis.. Symmetry (Basel).

[34] Hartigan J.A., Wong M.A. (1979). Algorithm AS 136: A K-means clustering algorithm.. Appl. Stat..

[35] Kassambara A., Mundt F. (2020). Extract and visualize the results of multivariate data analyses.. https://CRAN.R-project.org/package=factoextra.

[36] Wąż P., Bielińska-Wąż D., Nandy A. (2014). Descriptors of 2D-dynamic graphs as a classification tool of DNA sequences.. J. Math. Chem..

[37] Panas D., Wąż P., Bielińska–Wąż D., Nandy A., Basak S.C. (2017). 2D–dynamic representation of DNA/RNA sequences as a characterization tool of the Zika virus genome.. MATCH Commun. Math. Comput. Chem..

[38] Hou W., Pan Q., Peng Q., He M. (2017). A new method to analyze protein sequence similarity using dynamic time warping.. Genomics.

[39] Chen J., Hu C., Chen L., Tang L., Zhu Y., Xu X., Chen L., Gao H., Lu X., Yu L., Dai X., Xiang C., Li L. (2020). Clinical study of mesenchymal stem cell treatment for acute respiratory distress syndrome induced by epidemic influenza a (h7n9) infection: A hint for COVID-19 treatment.. Engineering (Beijing).

[40] Skowronski D.M., Chuang E.S.Y., Sabaiduc S., Kaweski S.E., Kim S., Dickinson J.A., Olsha R., Gubbay J.B., Zelyas N., Charest H., Bastien N., Jassem A.N., De Serres G. (2023). Vaccine effectiveness estimates from an early-season influenza A(H3N2) epidemic, including unique genetic diversity with reassortment, Canada, 2022/23.. Euro Surveill..

[41] Braga J.U., Ribeiro A.F. (2024). Biological, social, and healthcare factors for death due to influenza A(H1N1) during the 2009 epidemic in Brazil.. Rev. Saude Pub..

[42] Kim J.Y., Jeong S., Kim D.W., Lee D.W., Lee D.H., Kim D., Kwon J.H. (2024). Genomic epidemiology of highly pathogenic avian influenza a (h5n1) virus in wild birds in South Korea during 2021–2022: Changes in viral epidemic patterns.. Virus Evol..

[43] The R Project for Statistical Computing (2024). The R Project for Statistical Computing.. https://www.R-project.org/.

[44] Maechler M., Rousseeuw P., Struyf A., Hubert M., Hornik K. (2024). Cluster analysis basics and extensions.. Package ‘Cluster’..

[45] Beaudoin C.A., Kohli M., Salvage S.C., Liu H., Arundel S.J., Hamaia S.W., Lei M., Huang C.L.H., Jackson A.P. (2025). Isoform-specific N-linked glycosylation of NaV channel α-subunits alters β-subunit binding sites.. J. Gen. Physiol..

